# Magnetic Resonance Biomarkers in Neonatal Encephalopathy (MARBLE): a prospective multicountry study

**DOI:** 10.1136/bmjopen-2015-008912

**Published:** 2015-09-30

**Authors:** Peter J Lally, Shreela Pauliah, Paolo Montaldo, Badr Chaban, Vania Oliveira, Alan Bainbridge, Aung Soe, Santosh Pattnayak, Paul Clarke, Prakash Satodia, Sundeep Harigopal, Laurence J Abernethy, Mark A Turner, Angela Huertas-Ceballos, Seetha Shankaran, Sudhin Thayyil

**Affiliations:** 1 Centre for Perinatal Neuroscience, Imperial College London, London, UK; 2 University College Hospital, London, UK; 3 Medway NHS Foundation Trust, Kent, UK; 4 Department of Neonatology, Norfolk and Norwich University Hospitals Foundation Trust, Norwich, UK; 5 Department of Neonatology, University Hospitals Coventry and Warwickshire NHS Trust, Coventry, UK; 6 Department of Neonatology, Royal Victoria Infirmary, Newcastle, UK; 7 Liverpool Womens Hospital and Alderhey Hospital, Liverpool, UK; 8 Department of Neonatology, Children's Hospital of Michigan, Michigan, UK

**Keywords:** NEONATOLOGY, NEUROLOGY

## Abstract

**Introduction:**

Despite cooling, adverse outcomes are seen in up to half of the surviving infants after neonatal encephalopathy. A number of novel adjunct drug therapies with cooling have been shown to be highly neuroprotective in animal studies, and are currently awaiting clinical translation. Rigorous evaluation of these therapies in phase II trials using surrogate MR biomarkers may speed up their bench to bedside translation. A recent systematic review of single-centre studies has suggested that MR spectroscopy biomarkers offer the best promise; however, the prognostic accuracy of these biomarkers in cooled encephalopathic babies in a multicentre setting using different MR scan makers is not known.

**Methods and analysis:**

The MR scanners (3 T; Philips, Siemens, GE) in all the participating sites will be harmonised using phantom experiments and healthy adult volunteers before the start of the study. We will then recruit 180 encephalopathic infants treated with whole body cooling from the participating centres. MRI and spectroscopy will be performed within 2 weeks of birth. Neurodevelopmental outcomes will be assessed at 18–24 months of age. Agreement between MR cerebral biomarkers and neurodevelopmental outcome will be reported. The sample size is calculated using the ‘rule of 10’, generally used to calculate the sample size requirements for developing prognostic models. Considering 9 parameters, we require 9×10 adverse events, which suggest that a total sample size of 180 is required.

**Ethics and dissemination:**

Human Research Ethics Committee approvals have been received from Brent Research Ethics Committee (London), and from Imperial College London (Sponsor). We will submit the results of the study to relevant journals and offer national and international presentations.

**Trial registration number:**

Clinical Trials.gov Number: NCT01309711.

**Strengths and limitations of this study**

- First and largest multicentre prospective study examining the prognostic accuracy of cerebral MR biomarkers in neonatal encephalopathy.
- Robust optimisation, harmonisation and quality assurance of MR biomarkers.
- All the three major makers of MR scanners (Phillips, Siemens and GE) included.
- Only centres with access to 3 T MR scanners are included.

## Background

Neonatal encephalopathy occurs in approximately 1–2 per 1000 live births in high-income countries.^1–4^ The incidence in low-income and middle-income countries is much higher.^5^ Often, neonatal encephalopathy occurs unexpectedly following an otherwise uneventful pregnancy. Before the widespread implementation of the therapeutic hypothermia, 20–25% of the affected infants died in the first few days after birth, and up to 75% of the survivors developed significant lifelong disabilities^3^ ^6^ resulting in a substantial burden for the individual, their family and society.^7^

In the past 5 years, three major clinical trials of whole body or selective head cooling therapy^8–10^ showed significant reduction in death (risk ratio (RR)=0.8; 95% CI 0.7 to 0.9; p=0.005) and improvement in survival with normal neurological outcome (RR=1.5; 95% CI 1.2 to 1.9; p<0.001), after neonatal encephalopathy.^11^ The protective effect of therapeutic hypothermia persists into early childhood.^12^ Unfortunately, up to 50% of surviving infants still have adverse outcomes.^13^

For these reasons, there has been a renewed interest in other potential neuroprotectants, or adjunctive therapies, most of which have shown promising results in a preclinical stage or in pilot studies.^14^ ^15^

Unfortunately, evaluating clinical efficacy of these therapies is extremely challenging for a number of reasons. First, the sample sizes for an adequately powered clinical trial of an adjunct therapy will be exceedingly high. Second, the effects on long-term outcome are likely to be more subtle and may require several years of follow-up for adequate evaluation. Finally, the intervention and the outcome measures of these neuroprotectants are likely to be highly heterogeneous (eg, different protocols, dosage and timing of the intervention) unlike the three major cooling trials, thus reducing the chances of robust meta-analysis.

MR biomarkers of injury severity have the potential to overcome many of these challenging impediments to progress. In the past decade, such MR biomarkers have enhanced our ability to identify, assess the onset and quantify the features of brain injury soon after birth.^16^ These modalities may be valuable as surrogate outcome measures of the treatment effects of adjunct neuroprotective therapies.

While conventional and diffusion-weighted neonatal MRI is currently the main modality used for the assessment of injury and prediction of outcome after neonatal encephalopathy, it is considered subjective and requires considerable time and experience for interpretation. Measurements of basal ganglia or thalamic metabolites by proton MR spectroscopy (MRS), in particular the ratio of lactate/*N*-acetylaspartate (Lac/NAA), have been shown in meta-analyses to be the most accurate cerebral MR biomarkers for predicting medium-term adverse neurological outcome currently available.^16^ Unfortunately, these data come from a very few, small single-centre studies, using a variety of MR sequences on 1.5 T MR scanners,^17–19^ and their applicability over a wider range of centres is not known. There is very little information available on the use of these MRS biomarkers on babies who have received rescue hypothermic neuroprotective therapy and in general have lesser degrees of injury than in earlier cohorts; additionally, there is a lack of data at 3 T, which is expected to be the standard platform for neonatal neuroimaging in the next decade.

Metabolite peak area ratios are affected by pathophysiological changes in both NAA and Lac, which follow very different time courses and which can be variably and independently affected by neuroprotective therapies and intercentre scanner differences.^20^ Hence, despite the good reported prediction of outcome with metabolite ratios, absolute quantification of [NAA], even though technically more challenging to estimate than metabolite peak area ratios, may offer several advantages. NAA is the second most abundant amino acid in the nervous system, and is almost exclusively neuronal. Therefore, it has been used as a surrogate index of neuronal survival. NAA concentrations measured by ^1^H MRS are theoretically absolute, reproducible,^21^ should not vary with scanner magnetic field strength, investigatory centre^20^ and correlate well with NAA concentrations measured by high performance liquid chromatography.^22^ ^23^ Therefore, it is considered an ideal biomarker.

Smaller single-centre studies have also shown good correlation of reduced whole brain fractional anisotropy (FA; analysed by Tract Based Spatial Statistics—TBSS) with adverse outcomes at 2 years.^24^ ^25^ TBSS is an automated observer-independent method of aligning FA images from multiple subjects to make non-biased assessments of localised changes in the major white matter tracts,^25^ and therefore is a useful tool for evaluating treatment efficiencies of neuroprotective therapies on diffusion tensor-based biomarkers.

The current study aims to harmonise the acquisition, and to qualify the use of novel quantitative cerebral MRI and MRS biomarkers for accurate prognostication of medium-term adverse outcomes in term and near term infants after neonatal encephalopathy, across several tertiary neonatal centres in the UK, and worldwide.

## Methods and design

This is a prospective multicountry observational study, which will be conducted in three phases.

*Phase A*: MRS phantoms will be prepared (expected life span 2 years) and scanned in 3 T MR scanners at the participating centres. The intercentre variability of metabolite ratios and concentrations will be examined and any effects will be investigated.

*Phase B*: Five healthy adult volunteers will be scanned at each site. Adult volunteers should be of sound health, between 20 and 45 years of age, and not pregnant at the time of scans. The intercentre variability of metabolite measurements will be examined and any effects will be investigated further.

*Phase C*: Infants with neonatal encephalopathy of presumed hypoxic-ischaemic origin and born at term and who have had rescue hypothermic neuroprotection will be recruited. The index tests (cerebral MR biomarkers) and neurological outcome at 18–24 months of age will be performed independently, and masked to each other.

### Inclusion criteria

Term and near term infants (36–43 weeks gestation) with evidence of neonatal encephalopathy and treated with therapeutic hypothermia at the participating neonatal units will be eligible for recruitment.

### Exclusion criteria

- Life-threatening congenital malformations
- Syndromic infants, and babies with neurometabolic diseases
- Neurodevelopmental follow-up not possible
- Death/withdrawal of life support before completion of 72 h of therapeutic hypothermia
- Lack of parental consent or inability to scan within 2 weeks of birth

### Clinical assessment

All infants will have standard intensive care management for neonatal encephalopathy. Gestational age; birth weight and head circumference; the Apgar score at 1, 5 and 10 min; umbilical cord pH; base deficit and Lac or blood gas pH; base deficit; Lac within 1 h of birth will be recorded. The babies will have a cranial ultrasound scan on admission, at 24 h and 3–4 days for detection of malformations, bleeds, loss of normal tissue differentiation and evolving tissue injury.^26^ Amplitude-integrated EEG (aEEG) will be performed over the first 48 h, as part of standard clinical care. The aEEG background activity will be classified according to voltage criteria and by background pattern.^27^ We will perform a scored neurological assessment (National Institute of Child Health and Human Development scoring system^28^ ^29^), on admission, daily during the first 3 days and an additional more detailed assessment before discharge. Additional items will include: head circumference, head control, axial tone, limb tone, seizures, visual fixation and following, squint, hearing, feeding.

### MRI protocol

#### Acquisition

MRI will be performed between 4 and 14 days after birth (ie, after completion of therapeutic hypothermia) in all infants, following informed parental consent. Existing MR scanning protocols will be followed in the individual centres with regard to sedation and monitoring.

#### MRI

Detailed MR sequences for different scanners will be developed into individual MR taskcards at each site. Typical MR parameters on a Philips 3 T scanner are: T1-weighted three-dimensional (3D) magnetisation-prepared rapid gradient-echo (sagittal, repetition time (TR)=17 ms, echo time (TE)=4.6 ms, flip angle=13°, inversion time=1465 ms, 0.8×0.8×0.5 mm^3^ voxels), T2-weighted 2D turbo spin echo (axial, TE=130 ms, TR=8800 ms, 0.5×0.5×3 mm^3^ voxels) and diffusion tensor imaging (DTI; 32 direction 2D spin-echo echo-planar imaging, b=750 s/mm^2^, TR=7860 ms, TE=49 ms, 1.75×1.75×2 mm^3^ voxels, SENSE factor ×2).

#### Conventional MRI score

Each image will be reported systematically by an experienced neonatal neurologist blinded to the clinical history but not gestational age and age at scan. Each scan will be assessed for anatomy, established injury, atrophy, haemorrhage and ventriculomegaly. Based on the scoring system developed by Rutherford *et al*,^30^ brain tissue injury will be scored from 0 to 3 for basal ganglia/thalami, white matter and cortex (0=normal, 1=mild signal abnormality, 2=moderate signal abnormality, 3=severe signal abnormality) and the posterior limb of the internal capsule scored from 0 to 2, as normal, equivocal or abnormal. The presence of focal hemispheric lesions and their site will also be noted.

#### MRS metabolite peak area ratios

Single-voxel point-resolved spectroscopy (PRESS), 15×15×15 mm^3^ cubic voxel in the left thalamus, TR=2288 ms/TE=288 ms (16 averages with 8 phase cycles, water suppressed) and TR=2060 ms/TE=60 ms (8 averages with 8 phase cycles, water suppressed). Metabolite peak area ratios involving NAA, total choline, total creatine and Lac will be derived.

#### MRS quantification of [NAA]

Same thalamic voxel; additional PRESS scans: (1) TE=60 ms, TR=5000 ms, (8 averages with 8 phase cycles, water suppressed) (2) TE=60, 124, 205, 316, 495, and 1000 ms, each with TR=10s+TE, with one average of eight phase cycles, and without water suppression. NAA concentration will be derived from an assumed brain water concentration by comparing the T2-corrected fully relaxed NAA and brain water signals.

#### TBSS analysis

Whole-brain white matter FA with TBSS will be analysed using the Functional MRI of the Brain Software Library (FSL, V.4.1)^31^ and the Diffusion Tensor Imaging ToolKit (DTI-TK, V.2.3.1).^32^ Diffusion-weighted data will be corrected for motion and eddy current distortion, segmented to exclude extracerebral tissue, and used to reconstruct the diffusion tensor volume, with FSL. The diffusion tensor volumes from all the participants will be spatially normalised with an optimised pipeline in DTI-TK, prior to TBSS analysis.^33^ An FA threshold (>0.10) will be required to identify the major white matter tracts but to exclude peripheral tracts. TBSS will be used to assess the relationship between FA and outcome. The presence of group-wise FA differences with p<0.05, corrected for multiple comparisons using threshold-free cluster enhancement,^34^ will be considered statistically significant.

#### MR Spectroscopy Analysis

The raw data will be taken from MR scanners, postprocessed using software developed in-house, and LCModel (LCModel Inc, Oakville, Ontario, Canada, V.6.1) will be used to calculate metabolite peak area ratios and concentrations.

### Neurodevelopmental outcome

A standardised, validated and scorable neurological examination will be performed;^35^ the presence and type of cerebral palsy determined according to Gorter *et al*^36^ and severity classified using the Gross Motor Function Classification System (GMFCS). Neurodevelopmental outcome will be assessed using the Bayley Scales of Infant Development (BSID-III), by trained and certified examiners. Vision and audiometric evaluations^37^ will be collected alongside anthropometric data (weight, height and head circumference) as part of the follow-up evaluations. Infants will be tracked and undergo follow-up at network centres with evaluations at 18–24 months.

Outcome will be assessed according to the neurological examination score; the GMFCS level; and the cognitive, language and motor composite scores. These variables provide quantitative data that can be compared with MR measures.

Severe disability will be defined as any one of the following: Bayley III, both cognitive and language composite scores <70, GMFCS level 3–5, hearing impairment requiring hearing aids or blindness. Moderate disability will be defined as both cognitive and language composite scores between 70 and 84 and one or more of the following: GMFCS level 2, hearing impairment with no amplification or a persistent seizure disorder.

The outcomes will also be assessed by categorisation of these scores and by including mortality as an outcome. Adverse outcome will be defined as death or, in survivors, moderate or severe disability.

### Outcomes

The primary outcome is to examine the accuracy of quantitative cerebral MR biomarkers for predicting adverse 18–24-month neurodevelopmental outcome in infants who had neonatal encephalopathy treated with therapeutic hypothermia, in a multicentre setting.

The secondary outcomes are the intercentre variability of proton MRS measurements and the incremental benefits of quantitative MR biomarkers for predicting adverse outcomes when compared with conventional MR assessment and bedside assessments and investigations such as the Apgar score at 1, 5 and 10 min; umbilical cord pH; base deficit and Lac or blood gas pH; base deficit; Lac within 1 h of birth; abnormal aEEG (voltage criteria: upper margin <10 µV, lower margin <5 µV) at 48 h; neurological examination at discharge.

### Sample size calculation and data analysis

The primary outcome will be unfavourable outcome at 18–24 months (yes/no). Approximately 50% of the infants are expected to have an unfavourable outcome. Since this is binary, multivariable logistic regression will be used to develop risk prediction models. The predictors in these models will be: [NAA], Lac/NAA peak area ratio, white matter FA (all continuous); aEEG at 48h (categorical with three levels) and clinical examination at discharge (binary). Models will be validated using 10-fold cross-validation, and will be assessed with respect to calibration and discrimination. Calibration and discrimination will be measured using calibration slopes and receiver operating characteristic curves, respectively. Shrinkage techniques will be used to adjust for overfitting, thus improving calibration. Sensitivity and specificity will be calculated at selected cut-off points.

The sample size is calculated using the ‘rule of 10’,^2^ generally used to calculate the sample size requirements for developing prognostic models. The multivariable risk model will contain seven predictors that are modelled using nine parameters to examine the incremental benefits of various clinical, bedside (aEEG) and MR biomarkers (conventional MR reporting, MRS peak area metabolite ratios and [NAA]). Considering nine parameters, we require 9×10 adverse events, which suggest that a total sample size of 180 is required.

### Study approval

North London Research Ethics Committee has approved the MARBLE study (13HH1843). Imperial College London is the sponsor for the study. All participating centres have site-specific approval, and research contracts with Imperial College London will be setup before the start of the study.

### Consent

Informed written parental consent will be obtained as stipulated by the research ethics committee, prior to recruitment.

## Discussion

The use of a biomarker to measure the treatment effects of an intervention is complex. Wrong surrogate biomarker approaches can result not only in wasted effort and in resources, but can also cause patient harm. However, there are many instances where judicious use of imaging biomarkers has resulted in rapid bench to bedside translation and remarkable scientific progress, for example, in oncology.^38^

Furthermore, the use of cerebral MR biomarkers in multicentre studies can be particularly challenging. Hence, careful and robust optimisation of sequences and harmonisation of MR scanners and techniques is mandatory.

This study can help identify new biomarkers for accurate prognostication of long-term adverse outcomes in term and near term infants after neonatal encephalopathy.

Moreover, such surrogate cerebral MR biomarkers could give a beneficial contribution to future clinical trials. In fact, these biomarkers have far less variability than clinical neurodevelopmental outcome measurements, and hence the required sample sizes for equivalent risk reductions may be reduced. A further strength is that these outcomes can be measured soon after the perinatal intervention (ie, during the neonatal period itself) rather than after several years. For these reasons, they would enable direct comparison of multiple interventions using factorial trial designs with minimal resources and negate the influence of confounding factors. Thus, an approach using surrogate MR biomarkers has the potential to screen out the most promising therapies and to make ‘go’ or ‘no go’ decisions before phase III clinical trials.
